# Gene activation in *Caenorhabditis elegans* using the *Campylobacter jejuni* CRISPR-Cas9 feeding system

**DOI:** 10.1093/g3journal/jkac068

**Published:** 2022-04-04

**Authors:** Zhenhuan Luo, Wenyu Dai, Chongyang Wang, Qunshan Ye, Qinghua Zhou, Qin-Li Wan

**Affiliations:** Zhuhai Precision Medical Center, Zhuhai People’s Hospital (Zhuhai Hospital Affiliated with Jinan University), Jinan University, Guangzhou 510632, China

**Keywords:** *Campylobacter jejuni* Cas9, gene activation, feeding, *Caenorhabditis elegans*

## Abstract

Clustered regularly interspaced palindromic repeats-based activation system, a powerful genetic manipulation technology, can modulate endogenous gene transcription in various organisms through fusing nuclease-deficient Cas9 to transcriptional regulatory domains. At present, this clustered regularly interspaced palindromic repeats-based activation system has been applied to activate gene expression by microinjection manner in *Caenorhabditis* *elegans*. However, this complicated and time-consuming injection manner is not suitable for efficient and high-throughput gene regulation with clustered regularly interspaced palindromic repeats-Cas9 system. Here, we engineered a *Campylobacter jejun* clustered regularly interspaced palindromic repeats-Cas9-based gene activation system through bacteria feeding technique to delivering gene-specific sgRNA in *C. elegans*. It enables to activate various endogenous genes efficiently, as well as induce the corresponding phenotypes with a more efficient and labor-saving manner. Collectively, our results demonstrated that our novel d*Cj*Cas9-based activation feeding system holds great promise and potential in *C. elegans*.

## Introduction 

The clustered regularly interspaced palindromic repeats (CRISPR)-CRISPR-associated protein (CRISPR-Cas) system is most often based on the *Streptococcus pyogenes* Cas9 (*Sp*Cas9) nuclease, which is an RNA-guided genome editing tool for genetic manipulation in various organisms (Bevacqua *et al.* 2021; Farbiak *et al.* 2021; Yin *et al.* 2021). In addition to *Sp*Cas9, various Cas9 orthologs from different bacteria have been characterized and developed, such as *Neisseria meningitidis* Cas9 (*Nm*Cas9), *Staphylococcus aureus* Cas9 (*Sa*Cas9), *Francisella novicida* Cas9 (*Fn*Cas9), and *Campylobacter jejuni* Cas9 (*Cj*Cas9). To date, the CRISPR-Cas9 system has been widely used for introducing indels and generating knockouts in vitro and in vivo (Bevacqua *et al.* 2021; Farbiak *et al.* 2021; Yin *et al.* 2021). Moreover, when a missense substitution of the catalytic site was introduced to eliminate Cas9 nuclease activity that did not compromise its DNA binding activity, nuclease-deficient Cas9 (dCas9) was also able to upregulate RNA-guided transcription by fusing with trans-activators (Mali *et al.* 2013). Among these various Cas9 orthologs, *Cj*Cas9, a newer CRISPR endonuclease, exhibits several unique features, including its smallest size (984 amino acid residues) for easier delivery, distinct target recognition of the 5′-NNNNACA-3′ or 5′-NNNNRYAC-3′ sequence, unique triple-helix tracrRNA structure, and potential for contact with the nucleotide sequences in both DNA strands of the target (Zhang *et al.* 2021).

In *Caenorhabditis elegans*, the CRISPR-Cas9 system, especially *Sp*Cas9, has been widely used for gene editing and transcriptional regulation, either alone or in combination with transcriptional regulatory domains (Long *et al.* 2015; Wei *et al.* 2019). Very recently, we also developed a robust *Cj*Cas9-based transcription activation system, miniCAFE, which combines a nuclease-deficient *Cj*Cas9 (d*Cj*Cas9) and the tripartite transcriptional activator VP64-p65-Rta (herpes simplex virus-derived VP64 activator, the human NF-KB *p65* activator domain, and the Epstein-Barr-virus-derived R trans-activator, VPR), and then microinjected it into *C. elegans* to activate the expression of various genes (Zhang *et al.* 2021). Microinjection in *C. elegans* is a reliable, versatile, and frequently used method for delivering genetic constructs. However, microinjection requires a specialized micromanipulator and a skilled microinjection operator (Berkowitz *et al.* 2008), so it is not suitable for efficient and high-throughput gene disruption or regulation in the CRISPR-Cas9 system. In *C. elegans*, feeding-based RNAi is the most convenient and powerful method for silencing gene function by feeding animals with bacteria expressing dsRNA (Timmons *et al.* 2001). This “feeding” method has also been used for delivering guide-RNA to achieve CRISPR-Cas9-based gene disruptions (Liu *et al.* 2014). However, the CRISPR-based feeding system for gene activation has not been reported. Therefore, we engineered a CRISPR-Cas9-based gene activation system using a bacteria feeding technique to deliver gene-specific sgRNA to determine whether genes could be activated using a bacteria feeding-dependent CRISPR-Cas9 system.

## Materials and methods

### *Caenorhabditis elegans* strains and maintenance

Wild-type N2 (Bristol) was used as the wild-type strain [from the *Caenorhabditis* Genetic Center (CGC), University of Minnesota, USA]. Worms were grown and maintained on solid nematode growth medium (NGM) with the OP50 or HT115 *Escherichia* *coli* strains as food at 20°C.

### Plasmid construction

The plasmid pCAG-VPR-L1-*Cj*Cas9 D8A H559A was constructed by Rong Lab. To implement VPR-dCjCas9 as a transcriptional activator in *C. elegans*, the *dpy-30* promoter (a ubiquitous promoter) sequence was amplified from *C. elegans* N2 strain genomic DNA and cloned into a *SpeI*/*EcoRI*-digested pCAG-VPR-L1-*Cj*Cas9 D8A H559A plasmid to replace the CAG promoter. The VP64 sequence was amplified from the pCAG-VPR-L1-*Cj*Cas9 D8A H559A plasmid and cloned into the *NcoI*/*RsrII*-digested p*dpy-30*-VPR-L1-*Cj*Cas9 D8A H559A plasmid to replace the VPR activator sequence.

To generate a plasmid expressing gene-specific sgRNA driven by the T7 promoter, a T7 promoter-*lac operator*-target sequence-sgRNA^(F+E)^ scaffold-T7 terminator cassette was constructed and cloned into the L4440 vector to replace the convergently oriented T7 promoter fragment. The modified sgRNA scaffold [sgRNA^(F+E)^] was amplified from the PU6::*unc-119* sgRNA^(F+E)^ plasmid (Zhao *et al.* 2016), and the T7 promoter-*lac operator* and the T7 terminator sequences were directly added to the forward and reverse primers, respectively. The sequences of gene-specific sgRNAs of interest were inserted into the sgRNA plasmid by nested overlap PCR, and all sgRNAs were designed using *http://crispor.tefor.net/*. The guide RNA sequences and their primers are listed in Table 1. The full sequence of the T7 promoter-*lac operator*-target sequence- sgRNA^(F+E)^ scaffold-T7 terminator cassette is shown in Table 2.

**Table 1.** The sgRNAs and primers in this study.

Gene	Target genomic sequences (5′- 3′)	Primer sequences (5′–3′)	
*myo-2*	ATAAGAGTAGCAAAATGGCAGG	Forward	ATAAGAGTAGCAAAATGGCAGGGTTTAAGAGCTATGCTGGA
	AAGAGCAC	Reverse	CCTGCCATTTTGCTACTCTTATGGAATTGTTATCCGCTCAC
*lipl-4*	GATTTGCACTTCACATACACAC ACACACAC	Forward	GGATTTGCACTTCACATACACACGTTTAAGAGCTATGCTGGA
		Reverse	GTGTGTATGTGAAGTGCAAATCCGGAATTGTTATCCGCTCAC
*lipl-5*	AAGATAAGCTGTTTGGCGCTGT GAGGGTAC	Forward	GGAAGATAAGCTGTTTGGCGCTGTGTTTAAGAGCTATGCTGGA
		Reverse	ACAGCGCCAAACAGCTTATCTTCCGGAATTGTTATCCGCTCA
*aak-2*	AATATGTTCAGATGCTCGAGTG	Forward	GGAATATGTTCAGATGCTCGAGTGGTTTAAGAGCTATGCTGGA
	CAACGCAC	Reverse	CACTCGAGCATCTGAACATATTCCGGAATTGTTATCCGCTCACA
*pha-4*	TCTGTGCGAGACTATTAAAGTG	Forward	GGTCTGTGCGAGACTATTAAAGTGGTTTAAGAGCTATGCTGGA
	TTCAATAC	Reverse	CACTTTAATAGTCTCGCACAGACCGGAATTGTTATCCGCTCA
*spr-4*	AACCGAAAAGAGTGTTGGAGAC	Forward	GGAACCGAAAAGAGTGTTGGAGACGTTTAAGAGCTATGCTGGA
	GAAGATAC	Reverse	GTCTCCAACACTCTTTTCGGTTCCGGAATTGTTATCCGCTCACA
*pie-1* (sgRNA1)	CCATATCACTTTATGTGGCGTA	Forward	GGCCATATCACTTTATGTGGCGTAGTTTAAGAGCTATGCTGGA
	AAGAATAC	Reverse	TACGCCACATAAAGTGATATGGCCGGAATTGTTATCCGCTCACA
*pie-1* (sgRNA2)	AAAAATCTGTGAGTATCGCAAC	Forward	GGAAAAATCTGTGAGTATCGCAACGTTTAAGAGCTATGCTGGA
	GAAAGCAC	Reverse	GTTGCGATACTCACAGATTTTTCCGGAATTGTTATCCGCTCACA
*pie-1* (sgRNA3)	TTTTCTCCAATGTACTCGTACT	Forward	GGTTTTCTCCAATGTACTCGTACTGTTTAAGAGCTATGCTGGA
	CCAAGTAC	Reverse	AGTACGAGTACATTGGAGAAAACCGGAATTGTTATCCGCTCACA
*ges-1* (sgRNA1)	TTAAGCTTTTGGCATGAATACA	Forward	GGTTAAGCTTTTGGCATGAATACAGTTTAAGAGCTATGCTGGA
	GTGAACAC	Reverse	TGTATTCATGCCAAAAGCTTAACCGGAATTGTTATCCGCTCACA
*ges-1* (sgRNA2)	AAAATTTCAGTGGCCAGCACAA	Forward	GGAAAATTTCAGTGGCCAGCACAAGTTTAAGAGCTATGCTGGA
	ACACATAC	Reverse	TTGTGCTGGCCACTGAAATTTTCCGGAATTGTTATCCGCTCACA
*ges-1* (sgRNA3)	TTCTTGTAGTCAATAGATAGTG	Forward	GGTTCTTGTAGTCAATAGATAGTGGTTTAAGAGCTATGCTGGA
	CGAGACAC	Reverse	CACTATCTATTGACTACAAGAACCGGAATTGTTATCCGCTCACA
*rab-3* (sgRNA1)	TGTAGGCGCTTCTGTTAGAGAG	Forward	GGTGTAGGCGCTTCTGTTAGAGAGGTTTAAGAGCTATGCTGGA
	GAGAGCAC	Reverse	CTCTCTAACAGAAGCGCCTACACCGGAATTGTTATCCGCTCACA
*rab-3* (sgRNA2)	TGGTCGGATATTTGGGGATCAG	Forward	GGTGGTCGGATATTTGGGGATCAGGTTTAAGAGCTATGCTGGA
	AGAAGTAC	Reverse	CTGATCCCCAAATATCCGACCACCGGAATTGTTATCCGCTCACA
*chr IV* (NC)	AAAAGCTGGAAAACACACCAAC	Forward	GGAAAAGCTGGAAAACACACCAACGTTTAAGAGCTATGCTGGA
	ACACACAC	Reverse	GTTGGTGTGTTTTCCAGCTTTTCCGGAATTGTTATCCGCTCACA

**Table 2.** The full sequence of sgRNA cassette.

**The T7 promoter-*lac operator*-target sequence- sgRNA^(F+E)^ scaffold-T7 terminator cassette sequence (5′–3′)**
taatacgactcactataggggaattgtgagcggataacaattccGAACCCGTTGCCGAATACACGTTTAAGAGCTATGCTGGAAACAGCATAGCAAGTTTAAATAAGGCTAGTCCGTTATCAACTTGAAAAAGTGGCACCGAGTCGGTGCTTTTTTTctagcataaccccttggggcctctaaacgggtcttgaggggttttttg

### Microinjection and transgenic strains

To generate transgenic worms expressing the VPR-d*Cj*Cas9 or VP64-d*Cj*Cas9 fusion protein, microinjection was performed according to a standard protocol as previously described (Berkowitz et al. 2008). In brief, the relevant plasmids were injected into the gonads of young adult hermaphrodites. The DNA mixture for injection included 50 ng/μl P*dpy-30*::VPR-L1-*Cj*Cas9 D8A H559A or P*dpy-30*::VP64-L1-*Cj*Cas9 D8A H559A, 5 ng/μl P*myo-2*::GFP::H2B and 3 ng/μl P*myo-3*::*mCherry* (P*myo-2*::GFP::H2B and P*myo-3*::*mCherry* were used as the additional pharyngeal and body-wall fluorescence-bearing transgenic reporter plasmids, respectively). The VPR-d*Cj*Cas9 and VP64-d*Cj*Cas9 transgenic strains bearing the VPR-d*Cj*Cas9 or VP64-d*Cj*Cas9 expression plasmid and fluorescence reporter were obtained by microinjection as additional extrachromosomal arrays. To further generate stable transgenic worms, the extrachromosomal arrays were integrated into the chromosome by X-ray irradiation. The transgenic worms were back-crossed 4 times with the wild-type N2 strain before use.

### The sgRNA bacteria feeding assay

The empty vector or gene-specific sgRNA plasmids were transformed into HT115(DE3) chemically competent cells using CaCl_2_ transformation protocols. The gene-specific sgRNA sequences were verified by Sanger sequencing. All sgRNA bacteria feeding experiments were performed at 20°C using an RNAi-like feeding protocol (Wan *et al.* 2021). Briefly, in IPTG-supplemented plates, the VPR-d*Cj*Cas9 or VP64-d*Cj*Cas9 transgenic worms were fed HT115 bacteria carrying empty vector or gene-specific sgRNA plasmids starting at the L1 larval stage until the young adult stage. The sgRNA-fed worms were used for subsequent experiments.

### RNA extraction and quantitative RT-PCR

Worms were fed HT115 bacteria carrying empty vector or gene-specific sgRNA plasmids until age-synchronized young adults and were then collected with M9 buffer and washed several times. Worm pellets were resuspended using AG RNA^ex^ PRO reagent (Accurate Biology, Changsha, China). Total RNA was isolated by chloroform extraction and isopropanol precipitation. Afterward, 500 ng of total RNA was used for reverse transcription with a high-capacity cDNA transcription kit (RK20400, ABclonal, Wuhan, China). Quantitative real-time PCR was performed using SYBR Green Select Master Mix (RK21203, ABclonal, Wuhan, China) on a CFX96 real-time system (Bio Rad, CA, USA), and each experiment was repeated at least 3 times. Quantification of transcripts was normalized to the *cdc-42* gene, and results were computed using the 2^–ΔΔCt^ method. *P*-values were calculated using the 2-tailed Student’s *t*-test. The primers used in this study are shown in Table 3.

**Table 3.** The primers used in qRT-PCR.

Gene		Primer sequences (5′–3′)
GFP(S65C)	Forward	TCTGTCAGTGGAGAGGGTGA
	Reverse	GACAAGTGTTGGCCATGGAAC
*lipl-4*	Forward	AAAACAAGACCTGGAAGAAACG
	Reverse	ATAAACTTGGCTGGCTGCAT
*lipl-5*	Forward	TCAGGATGTTGTGGGAAGCC
	Reverse	GGCCATGTTACGTTTGTTTTCC
*aak-2*	Reverse	ATAGGAAGGAGGACGGTGGT
	Forward	GTCCTTGCGTTCCTTTCTTGAC
*pha-4*	Forward	GCCAATTTCATGCAAGGAGG
	Reverse	GCCAGTGGTAAAACCAAGAGGT
*spr-4*	Forward	AATTGTTCACAAGGTCAAGA
	Reverse	CCAACACATTCCTTCAAATA
*pie-1*	Forward	TTCAATGATTCGCTGTCGTCC
	Reverse	CGCGTTTTGTATTCTGTGTGCTTA
*ges-1*	Forward	TGCTAAAACCGGAGTTCCCC
	Reverse	GCAGTTCGTTCCAGAAAGCG
*rab-3*	Forward	TGACGATGTGTTGGAGGCATT
	Reverse	CCAGCCGCCATTCGACTT
*cdc-42*	Forward	CTGCTGGACAGGAAGATTACG
	Reverse	CTCGGACATTCTCGAATGAAG
*acl-1*	Forward	GCTGGACGTGATCTTACTGATTACC
	Reverse	GTAGCAGAGCTTCTCCTTGATGTC
*csq-1*	Forward	AACTGAGGTTCTGACCGAGAAG
	Reverse	TACTGGTCAAGCTCTGAGTCGTC

### Fluorescence microscopy and image analyses

To assess the fluorescence intensity of *myo-2*::GFP, we fed VPR-d*Cj*Cas9 and VP64-d*Cj*Cas9 transgenic worms HT115 bacteria carrying an empty vector or sgRNA targeting the *myo-2* promoter. We mounted the worms on 2% agar pads after they were anesthetized using M9 buffer containing NaN_3_ (50 mM) and then observed fluorescence using a Nikon Ti2-U (Zhang *et al.* 2021), The GFP fluorescence intensity of each worm was analyzed using ImageJ as previously described. Each experiment used at least 30 animals. P values were calculated using the 2-tailed Student’s *t*-test.

### Lifespan assay

All lifespan experiments were performed using standard protocols, as previously described (Zhang *et al.* 2021). Briefly, synchronized L1 animals were fed HT115 bacteria carrying empty vector or gene-specific sgRNA plasmids until the L4 larval stage. Then, approximately 100–120 young adult worms were transferred to IPTG-supplemented plates to conduct survival analyses. The corresponding HT115 bacteria were seeded before use. To prevent progeny production, 10 μM *5-fluoro-2′-deoxyuridine* (FUDR, Sigma) was also added. Death events were scored daily, and experiments were repeated at least twice. The SPSS package was used for statistical analysis, and the log-rank (Mantel–Cox) method was used to determine the significant difference. A *P*-value < 0.05 was considered statistically significant.

### Oil Red O staining and quantification

Synchronized worms were collected in M9 buffer. Then, Oil Red O (ORO) staining was conducted according to standard protocols (Zhang *et al.* 2021). ORO-stained animals were mounted onto 2% agar pads and imaged using a Nikon Ti2-U fluorescence microscope at 20× magnification. The mean intensity values in arbitrary units (*a.u.*) of per worm were graphed by using Image-Pro-Plus processing software and the lipid levels were determined using GraphPad Prism using a 2-tailed Student’s *t*-test. At least 30 animals were used in each experiment. The experiment was repeated at least twice.

## Results and discussion

**Fig. 1.**
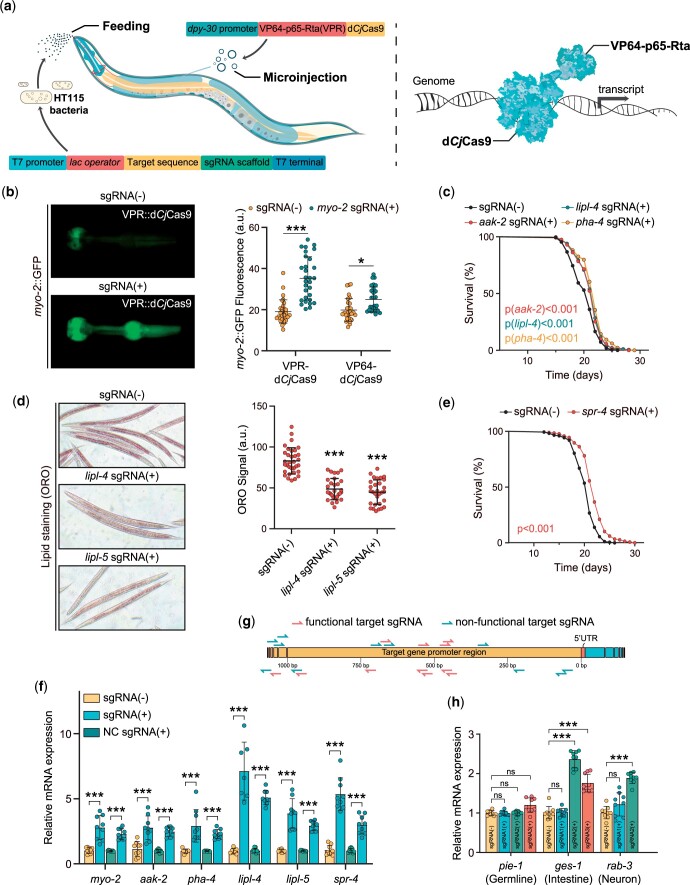
Transcriptional activation of multiple targeted genes and corresponding phenotype induction in *C. elegans* using the *C. jejuni* CRISPR–Cas9 activation feeding system. a) Schematic of the VRP-d*Cj*Cas9 activation feeding system. VPR-d*Cj*Cas9 transgenic worms were generated by injection of the P*dpy-30*::VPR-d*Cj*Cas9 plasmid. By delivering the engineered sgRNA plasmid containing the gene-specific gRNA sequence through bacterial feeding, the VRP-d*Cj*Cas9 activator can activate gene expression in *C. elegans*. b) P*myo-2*::GFP fluorescence in the pharyngeal region of the VPR-d*Cj*Cas9 and VP64-d*Cj*Cas9 strains bearing the P*myo-2*::GFP::H2B transgene in the presence or absence of *myo-2*-specific sgRNA targeting the promoter region. c) Survival analyses of the VPR-d*Cj*Cas9 worms in the presence or absence of *aak-2-*, *pha-4-*, or *lipl-4*-specific sgRNA targeting the promoter region. d) Fat levels in the intestinal region of the VPR-d*Cj*Cas9 strains in the presence or absence of *lipl-4-* or *lipl-5*-specific sgRNA targeting the promoter region. e) Survival analyses of the VPR-d*Cj*Cas9 worms in the presence or absence of the *spr-4*-specific sgRNA targeting the promoter region. f) Relative mRNA expression of target genes in the VPR-d*Cj*Cas9 worms in the presence or absence of the specific sgRNA targeting the target gene promoter region. sgRNA (−), empty vector L4440; sgRNA (+), gene-specific sgRNA plasmid; NC sgRNA, negative control sgRNA plasmid. g) Schematic of the distribution of all designed sgRNAs in the promoter regions of the target genes. h) Relative mRNA expression of tissue-specific genes, including *pie-1*, *ges-1*, and *rab-3*, in the VPR-d*Cj*Cas9 worms in the presence or absence of the specific sgRNA targeting the target gene promoter region. In (b) and (d), data are displayed as the mean ± SD*, n *≥* *30. In (c) and (e), lifespan analyses were performed using the Kaplan–Meier plotter, and the p value was determined by the log-rank test. The detailed lifespan values are listed in Table 5. In (f) and (h), the data are displayed as the mean ± SD of 3 independent experiments. In (b), (d), (f), and (h), ***P < 0.001, *P < 0.05, ns, not significant (Student’s *t*-test).

In this study, considering efficiency and specificity, we used *Cj*Cas9-based transcriptional activator plasmid pCAG-VPR-L1-*Cj*Cas9 D8A H559A {containing a DNase-dead *Cj*Cas9 [d*Cj*Cas9 (D8A H559A)] and a strong synthetic VPR activator} to conduct our examination. To implement VPR-d*Cj*Cas9 as a transcriptional activator in *C. elegans*, we replaced the CAG promoter with a ubiquitous (*dpy-30*) promoter (Fig. 1a). Afterward, we constructed transgenic animals ubiquitously and stably expressing the VPR-d*Cj*Cas9 fusion protein by microinjecting the P*dpy-30*::VPR-d*Cj*Cas9 plasmid into worm gonads.

To ensure efficient transcription of sgRNA in HT115 bacteria, a T7 promoter was used to drive an sgRNA cassette, including a target-specific sequence and a structurally modified sgRNA [sgRNA ^(F+E)^; Zhao *et al.* 2016] scaffold sequence. A *lac operator* was added between the T7 promoter and the target sequence to ensure sgRNA expression induced by *isopropyl-beta-D-galactoside* (IPTG). Furthermore, a T7 RNA polymerase terminator was added to the *3′* end of the scaffold to ensure the correct termination of sgRNA transcription (Fig. 1a). Afterward, the sgRNA cassette was cloned into the L4440 vector to replace the convergently oriented T7 promoter fragment and then transformed into HT115 bacteria (Fig. 1a).

We fed VPR-d*Cj*Cas9 transgenic worms grown on plates containing IPTG using HT115 bacteria carrying gene-specific sgRNA for the promoter or empty vector L4440 (without the sgRNA cassette as a control) from L1 larvae to the young adult stage. The worms fed sgRNA were then collected to determine transcriptional levels and corresponding phenotype analyses to assess the efficacy of our VPR-d*Cj*Cas9 activation feeding system. Quantification of transcripts was normalized using the housekeeping gene *cdc-42* (Hoogewijs *et al.* 2008), whose mRNA levels were not affected by sgRNA-bacteria, which was verified using other internal reference genes (*acl-1* and *csq-1*; Hoogewijs *et al.* 2008; data not shown). In addition, to quantify transcripts, we used both empty vector L4440 and an sgRNA targeting an intergenic sequence as the negative control (Fig. 1f).

During injection of plasmid P*dpy-30*::VPR-d*Cj*Cas9, P*myo-2*::GFP::H2B was used as a coinjection maker to ensure successful microinjection. To test the capacity of the VPR-d*Cj*Cas9 feeding system, we first examined whether our VPR-d*Cj*Cas9 activator could up-regulate the pharyngeal GFP fluorescence expression by sgRNA targeting the *myo-2* promoter. We fed the VPR-d*Cj*Cas9 transgenic worms HT115 bacteria carried with *myo-2*-specific sgRNA for promoter. We observed a significant increase in the fluorescence intensity of pharyngeal-GFP (Fig. 1b) and mRNA expression level when compared with the VPR-d*Cj*Cas9-only control worms (Fig. 1f), suggesting that the VPR-d*Cj*Cas9 feeding system could be used to activate the target gene in *C. elegans*.

Given the ability of the VPR-d*Cj*Cas9 system to upregulate reporter gene expression, we speculated that it might also induce endogenous gene transcription in *C. elegans*. Therefore, we used the VPR-d*Cj*Cas9 feeding system to activate a series of endogenous genes and conducted phenotype analyses.

First, we chose *aak-2* and *pha-4* to examine the ability of the VPR-d*Cj*Cas9 system to induce endogenous gene transcription. Both the AMPK α-catalytic subunit AAK-2 and the human FoxA transcription factor ortholog PHA-4 play important roles in lifespan regulation (Greer *et al.* 2007; Panowski *et al.* 2007). By delivering specific sgRNA targeting the *aak-2* or *pha-4* promoter using bacterial feeding, we observed a significant extension in mean lifespan (Fig. 1c) and greatly increased mRNA levels of the target (Fig. 1f), compared with control. Furthermore, we used lipid metabolism-regulating genes (*lipl-4* and *lipl-5*) to assess our VPR-d*Cj*Cas9 system. The lysosomal acid lipases LIPL-4 and LIPL-5 have been reported to regulate lipid storage and longevity in *C. elegans* (Folick *et al.* 2015; Buis *et al.* 2019). Consistent with previous findings, using ORO staining, we found that the fat storage levels of *C. elegans* were significantly decreased when the VPR-d*Cj*Cas9 feeding system was used to target the *lipl-4* or *lipl-5* promoter, respectively (Fig. 1d). Moreover, up-regulation of the mRNA expression levels of *lipl-4* or *lipl-5* was also detected (Fig. 1f). Similar to *aak-2* and *pha-4*, we also observed a significant lifespan extension when targeting *lipl-4* promoter (Fig. 1c). Recently, researchers have used engineered dCas9::VP46 by microinjection manner, to successfully induce the mammalian REST orthologue *spr-4* transcriptional activation and lifespan extension in worms (Zullo *et al.* 2019). Consistently, using our VPR-d*Cj*Cas9 feeding system with *spr-4*-specific-sgRNA, we also observed upregulation of the mRNA level of *spr-4* with corresponding lifespan extension (Fig. 1, e and f). Altogether, these results suggest that the feeding-based VPR-d*Cj*Cas9 activation system enables efficient activation of endogenous genes in *C. elegans*.

Compared with the traditional microinjection activation system, our VPR-d*Cj*Cas9 feeding system, although not as efficient as microinjection systems, is more efficient and less labor-intensive than injection systems (Zhang *et al.* 2021). Another gene activation system using herpes simplex virus-derived VP64 as an activator has been reported in *C. elegans* (Long *et al.* 2015; Zullo *et al.* 2019). To compare the activation efficiency of VP64 and the VPR activator, we generated VP64-d*Cj*Cas9 transgenic worms and then assessed the ability of VP64 and the VPR activator to induce gene expression using the same sgRNA targeting the *myo-2* promoter by feeding manner. Compared with the control worms, we observed a significant increase in the fluorescence intensity of pharyngeal-GFP both in VPR-d*Cj*Cas9 and VP64-d*Cj*Cas9 worms, but those of VP64-d*Cj*Cas9 worms were weaker, suggesting that the activation efficiency of the VPR activator was obviously stronger than that of VP64 (Fig. 1b).

Furthermore, to determine the activation efficiency of our VPR-d*Cj*Cas9 feeding system in different tissues, we attempted to activate tissue-specific genes including *pie-1* (germline), *ges-1* (intestine), and *rab-3* (neuron), by delivering specific-sgRNAs of targets through bacteria feeding. We found significant up-regulation of the mRNA levels of *ges-1* and *rab-3*, but not *pie-1*, by feeding the gene-specific sgRNA bacteria (Fig. 1h). These results suggest that our feeding-based VPR-d*Cj*Cas9 system exhibits different efficiencies in different tissues, with a high activation efficiency in the intestine, pharynx (confirmed by *myo-2*) and neuron, but poor efficiency (even nonfunctional) in the germline.

As so far, a modular and flexible platform for gene activation in vivo has been built using the dCas9 protein combined with trans-activator domains (Böhm *et al.* 2020; Chiarella *et al.* 2020). In this study, we developed a CRISPR-*Cj*Cas9-based system to efficiently activate transcription through bacteria feeding to deliver gene-specific sgRNA in *C. elegans*, which is cost-effective and efficient. However, it is worth noting that targeting either the different genes or the same gene in different sites using our VPR-d*Cj*Cas9 feeding system exhibited markedly different activation efficiencies (Table 4). Therefore, a preliminary screening to obtain suitable and efficient sgRNAs is required before corresponding studies using the VPR-dCjCas9 feeding system. To better select the functional sgRNAs, we analyzed the distribution of all designed sgRNAs in the promoter region of the different target genes and found that the functional sgRNAs were primarily distributed between 400 and 700 bp upstream of the *5′UTR* of target genes (Fig. 1g). Moreover, a previous study indicated that over-expression SID-1 and SID-2 transgenic worms could be selected as a powerful genetic background to increase gRNA uptake (Liu *et al.* 2014). In further study, we will attempt to use the SID-1 and SID-2 over-expression transgenic background to optimize our VPR-d*Cj*Cas9 feeding system. Furthermore, the expression of most genes could be regulated by transcription factors or epigenetic modifiers. Therefore, combining d*Cj*Cas9 with other functional domains, such as an epigenetic modifier, would greatly expand the applicability of our CRISPR-Cas9 feeding system in *C. elegans*. Overall, this novel d*Cj*Cas9-based feeding system may hold great promise for genome editing, transcriptome modulation, and other applications in *C. elegans*.

**Table 4.** The sgRNA screening of multiple targeted genes.

Target gene		Target sequences (5′–3′)	Fold change
*myo-2*	sgRNA1	GGGGATGTCACAATAAAACGTC CACAGCAC	1.30
	sgRNA2	AAATGAAGAATCGGGGGCACTG ATTAACAC	**3.98**
	sgRNA3	ATAAGAGTAGCAAAATGGCAGG AAGAGCAC	**2.72**
*myo-3*	sgRNA1	GCGAATATAATGGAATATAATG GATCACAC	1.49
	sgRNA2	AGCAACAGAATCTATACAACAC GCACACAC	1.09
	sgRNA3	CTATTTTCACTTCGGGAGCCCA ACCAACAC	1.38
*aak-2*	sgRNA1	AATATGTTCAGATGCTCGAGTG CAACGCAC	**3.14**
	sgRNA2	TTTTGTTTGGCGAGAGGAAGGA AAAAGCAC	1.77
	sgRNA3	AAAGGTTTGCTGTGTAAATAGA ACAAGTAC	1.06
*akt-1*	sgRNA1	GTTGGAAAACAAAAAACCACAG ACGAACAC	1.31
	sgRNA2	GTGACCCTATTTCAGCGGAAAC AAGAACAC	**1.92**
	sgRNA3	TGGAAAACAAGATTATTTCTAC GATAACAC	1.21
*lipl-4*	sgRNA1	CGTAGTCTGGCTTCTCCCTCAT CTTAATAC	1.09
	sgRNA2	GATTTGCACTTCACATACACAC ACACACAC	1.83
	sgRNA3	ATGACAACTGAAATTGCAGGAG CATCATAC	**8.69**
*lipl-5*	sgRNA1	TGCCGGGGAAGAGTGTGGTGAA CGTAGTAC	1.34
	sgRNA2	AAGATAAGCTGTTTGGCGCTGT GAGGGTAC	**2.60**
	sgRNA3	CACAGTACCCTTAATGCGTCGA TAAAGTAC	**3.70**
*cps-6*	sgRNA1	ATTTCCCGGTGATTGCCGAATC GGAGACAC	1.87
	sgRNA2	AGGTAGAAGTTGCCAACAAAAC CGACACAC	1.43
	sgRNA3	GCCAGCGACAAATTGCTCCACG AGTAGTAC	1.57
*pha-4*	sgRNA1	ACCACTATACGATTCTGGGTCA CTAAACAC	0.92
	sgRNA2	AAGGGAATGGACATAGAGACGC AGTAACAC	1.58
	sgRNA3	TCTGTGCGAGACTATTAAAGTG TTCAATAC	**3.00**
*spr-4*	sgRNA1	TCAGACACAGGGTCAACGTAAT TAAAGTAC	1.23
	sgRNA2	GTCGGCTAATGGGCGGTTAGAA ACTAGCAC	**2.46**
	sgRNA3	AACCGAAAAGAGTGTTGGAGAC GAAGATAC	**6.05**
*pie-1*	sgRNA1	TCAGACACAGGGTCAACGTAAT TAAAGTAC	0.99
	sgRNA2	GTCGGCTAATGGGCGGTTAGAA ACTAGCAC	1.00
	sgRNA3	AACCGAAAAGAGTGTTGGAGAC GAAGATAC	1.20
*ges-1*	sgRNA1	TCAGACACAGGGTCAACGTAAT TAAAGTAC	1.02
	sgRNA2	GTCGGCTAATGGGCGGTTAGAA ACTAGCAC	**2.36**
	sgRNA3	AACCGAAAAGAGTGTTGGAGAC GAAGATAC	**1.76**
*rab-3*	sgRNA1	TCAGACACAGGGTCAACGTAAT TAAAGTAC	1.22
	sgRNA2	GTCGGCTAATGGGCGGTTAGAA ACTAGCAC	**1.89**

Bold values: p < 0.05

## Data availability

The *C. elegans* strains and plasmids are available upon request. Table 1 contains the sgRNAs and primers sequence. Table 2 contains the full sequence of feeding sgRNA cassette. Table 3 contains the primers used in qRT-PCR. Table 4 contains the sgRNA screening data of all candidate genes. Table 5 contains the statistics data of all lifespan assays. The authors affirmed that all data necessary for confirming the conclusions of the article are present within the article.

**Table 5.** The lifespan data and statistics.

Figure	Strains	Treatments	Mean lifespan ± SEM (d)	*P*-value VS sgRNA (−)	Change in mean lifespan (%)	*N*
**Fig. 1c**	**VPR-d*Cj*Cas9**					
	EXP.1	sgRNA (−)	20.262 ± 0.160	—	—	229
		*aak-2* sgRNA (+)	21.593 ± 0.168	<0.001	6.57	214
		*pha-4* sgRNA (+)	21.432 ± 0.177	<0.001	5.77	192
		*lipl-4* sgRNA (+)	21.821 ± 0.218	<0.001	7.69	117
	EXP.2	sgRNA (−)	20.291 ± 0.162	—	—	206
		*aak-2* sgRNA (+)	21.208 ± 0.166	<0.001	4.52	178
		*pha-4* sgRNA (+)	21.680 ± 0.180	<0.001	6.85	181
		*lipl-4* sgRNA (+)	21.284 ± 0.235	<0.001	4.89	95
	EXP.3	sgRNA (−)	19.807 ± 0.151	/	/	187
		*aak-2* sgRNA (+)	21.410 ± 0.177	<0.001	8.09	173
		*pha-4* sgRNA (+)	21.241 ± 0.165	<0.001	7.24	170
		*lipl-4* sgRNA (+)	21.039 ± 0.266	<0.001	6.22	77
**Fig. 1e**	**VPR-d*Cj*Cas9**					
	EXP.1	sgRNA (−)	20.554 ± 0.223	—	—	195
		*spr-4* sgRNA (+)	21.743 ± 0.195	<0.001	5.78	191
	EXP.2	sgRNA (−)	20.444 ± 0.205	—	—	178
		*spr-4* sgRNA (+)	22.227 ± 0.221	<0.001	8.72	181
	EXP.3	sgRNA (−)	20.217 ± 0.177	—	—	180
		*spr-4* sgRNA (+)	22.151 ± 0.220	<0.001	9.57	166

QZ, Q-LW, and ZL designed the study, ZL, WD, CW, and QY conducted the experiments. Q-LW, ZL, and CW analyzed the data. ZL and WD wrote the manuscript. QZ and Q-LW reviewed and edited the manuscript.

## Funding

This project has been supported by the National Key R&D Program of China (2018YFC2002000), the Program of Introducing Talents of Discipline to Universities (111 Project, no. B16021), the Natural Science Foundation of Guangdong Province, China (2018A0303131003), the Science and Technology Plan Project of Guangzhou, China (202002030021), the National Natural Science Foundation of China (82001465), and the Guangdong Basic and Applied Basic Research Foundation (2020A1515111026).

## Conflicts of interest

None declared.
